# miRNA–mRNA Associated With Survival in Endometrial Cancer

**DOI:** 10.3389/fgene.2019.00743

**Published:** 2019-08-20

**Authors:** Xiaofeng Xu, Tao Liu, Yijin Wang, Jian Fu, Qian Yang, Jun Wu, Huaijun Zhou

**Affiliations:** ^1^Department of Gynecology, The Affiliated Drum Tower Hospital of Nanjing University Medical School, Nanjing, China; ^2^Medical College, Nanjing University, Nanjing, China; ^3^Medical College, Southeast University, Nanjing, China; ^4^Department of Gynecology, Suqian People’s Hospital of Nanjing Drum Tower Hospital Group, Suqian, China; ^5^Department of Gynecology and Obstetrics, The Pukou Hospital of Nanjing, The Fourth Affiliated Hospital of Nanjing Medical University, Nanjing, China

**Keywords:** endometrial carcinoma, miR-497, EMX1, survival, TCGA

## Abstract

Although various factors may contribute to its initiation and progression, the etiology and prognostic factors of endometrial carcinoma (EC) remains not fully understood. We sought to understand the role of changes in transcriptome during the progress of EC by exploring public datasets. The aberrant expression characteristics of EC based on RNA-Seq and miRNA-seq data from The Cancer Genome Atlas (TCGA) were analyzed. Kaplan–Meier analysis was performed to assess the relationship between differently expressed genes (DEGs) and patient survival. As a result, 320 out of 4,613 differently expressed mRNAs (DE mRNAs) and 68 out of 531 differently expressed miRNAs (DE miRNAs) with a significantly poorer survival were determined. We predicted eight paired DE miRNAs and DE mRNAs through TargetScan. Patients with three out of the eight paired low rate of miRNA/mRNA (miR-497/EMX1, miR-23c/DMBX1, and miR-670/KCNS1) expression had a significantly poorer survival. Furthermore, the simultaneous presence of these selected low miRNA/mRNA pairs occurred in most patients and resulted in a significantly poorer survival rate. Luciferase reporter assay identified that EMX1 was a direct target of miR-497. Both low expression of miR-497 and overexpression of EMX1 were significantly associated with more advanced clinicopathologic characteristics (stage, tumor status, grade, and histology) besides survival (all P values < 0.05). Multivariate analysis also demonstrated that miR-497 remained an independent prognostic variable for overall survival. In summary, we identified that a series of DE mRNAs and miRNAs, including eight paired DE miRNAs and mRNAs, were associated with survival in EC. Clinical evaluation of downregulated miR-497 and paired upregulated EMX1 confirmed the value of our prediction analysis. The simultaneous presence of low rate of these selected low miRNA/mRNA pairs (miR-497/EMX1, miR-23c/DMBX1, and miR-670/KCNS1) might have a better prediction value. Therefore, further studies are required to validate these findings.

## Introduction

Endometrial cancer (EC) is one of the most prevalent gynecological malignancies in the word (Siegel et al., 2018). The incidence has increased by 1.2% per year from 2005 to 2014, along with the mortality rates, which similarly increased during this period (Smith et al., 2018). Importantly, EC is one of only two common cancers that defy the general trend of improvement in morbidity and mortality, with a worse survival rate today than in the 1970s (Siegel et al., 2018). The American Cancer Society (ACS) estimates that 63,230 women will be diagnosed with EC and that more than 11,000 women will die from this disease in 2018 (Siegel et al., 2018). Clearly, the burden of EC is increasing in the USA and worldwide, hence increasing the need to investigate its causes and improve prevention, early diagnosis, and treatment (Binder and Mutch, 2014). Although various endocrine, genetic, and external factors may contribute to its initiation and progression, the etiology and prognostic factors of endometrial carcinoma remain not fully understood.

Previous analysis of the human genome revealed that while ∼85% is transcribed, only ∼1% is protein-coding mRNA (Dunham et al., 2012; Djebali et al., 2012; Dykes and Emanueli, 2017). With time, more and more attention was being paid to the research of non-coding RNAs, including microRNAs (miRNAs), which have important regulatory roles in cancer cellular biology. Studies demonstrated that mature miRNAs can regulate the expression of their target genes by imprecise complementation to the 3′-untranslated regions (UTRs), 5′-UTRs, and even coding sequences of the mRNAs to repress their translation (Ambros, 2004; Duursma et al., 2008; Sacco and Masotti, 2012). Srivastava et al. reviewed the amount of miRNAs differentially expressed in EC versus normal endometrial tissue, including the increased expression of miR-9, miR-92a, miR-141, miR-182, miR-183, miR-186, miR-200a, miR-205a, miR-222, miR-223, miR-410, miR-429, miR-449, and miR-1228 and downregulation of miR-99b,miR-143, miR-145, miR-193b, and miR-204 (Srivastava et al., 2017). Numerous miRNAs could regulate EC cells by silencing their target genes (Zhou et al., 2012; Chen et al., 2016). Though expression patterns of several miRNAs were found to be associated with the International Federation of Gynaecology and Obstetrics (FIGO) stage, grade, relapse, and nodal metastases in EC (Torres et al., 2013; Canlorbe et al., 2016; Srivastava et al., 2017), few studies focused on the relationship between both miRNAs and their target genes with patient survival.

The Cancer Genome Atlas (TCGA) studies have defined the molecular genetic landscape of EC and highlighted the molecular genetic diversity of both endometrioid and non-endometrioid cancers (Burk et al., 2017). Recently, accompanied by the advent era of sharing information, more and more cancer researches including EC were carried out based on TCGA database (Dellinger et al., 2016; Reyes et al., 2017; Wang et al., 2018; Wu and Zhang, 2018). However, the previous studies mainly focused on the single factor, such as differently expressed mRNAs (DE mRNAs), differently expressed miRNAs (DE miRNAs), or differently expressed long non-coding RNAs (DE lncRNAs) to find the potential etiology and prognostic factor of tumor.

In this study, considering the silence effect to their target genes of miRNAs, we performed survival analysis on both DE mRNAs and DE miRNAs comparing EC and normal samples from TCGA database. As a result, we revealed a group of DE mRNAs and DE miRNAs associated with survival. Furthermore, we identified that low expression of miR-497 and overexpression of its potential target gene Empty Spiracles Homeobox 1 (EMX1) were both related to more advanced clinicopathologic characteristics.

## Methods

### Data Collection

Expression profiles of RNA-Seq and miRNA-seq for TCGA-UCEC project were downloaded from TCGA official website (https://cancergenome.nih.gov) in July 2018. RNA expression data of 543 EC cases and 35 normal cases were downloaded from the database, while miRNA expression data of 539 EC and 33 normal samples were included. The corresponding clinical information was downloaded from http://www.cbioportal.org. Data were collated and extracted for analysis.

### Identification of Differently Expressed Genes (DEGs)

We applied the expression profiles to the edgeR package in R language and calculated for differential expression between tumor and normal group samples after normalization and filtration. The adjusted *P* values (adj. *P*) were applied to correct the false-positive results. Then the significant DEGs (adj. *P* < 0.05 and fold-change value larger than 2) were selected out for the next step analysis.

### Survival Analysis

In this study, survival analysis refers to the overall survival (OS) Kaplan–Meier estimate. We performed Kaplan–Meier analysis (R package “survival”) to assess survival and relapse difference across cases with DEGs. For each gene, patients were divided into high-expression group and low-expression group according to the median expression level. Based on the survival curves of each group, these upregulated and downregulated DEGs with a poorer survival rate were within our consideration. *P* value < 0.05 was set as the cutoff point.

### Functional and Pathway Enrichment Analysis of DEGs

The Database for Annotation, Visualization and Integrated Discovery (DAVID, https://david.ncifcrf.gov/) is a biological database regularly used to facilitate functional annotation and pathway analysis. Kyoto Encyclopedia of Genes and Genomes (KEGG) pathway analysis aims to identify and visualize significantly enriched pathways of molecular interactions, reactions, and relations. Gene Ontology (GO) analysis uses hypergeometric tests to perform enrichment analysis on gene sets (Falcon and Gentleman, 2007). We uploaded selected DEGs associated with survival to DAVID to perform KEGG pathway and GO enrichment analysis. The human genome was selected as the background list parameter, and *P* value < 0.05 was set as the cutoff point.

Gene Set Enrichment Analysis (GSEA) is a computational method that determines whether a predefined set of genes shows significant differences between two biological phenotypes under study (Subramanian et al., 2005). Gene set permutations were performed 1,000 times for each analysis. The nominal *P* value (NOM *P* value) and normalized enrichment score (NES) were used to sort the pathways enriched in each phenotype.

### Prediction of DE miRNA-Targeted DE mRNAs Associated With OS

In order to improve the validity of our search results, we further excavated the relationship between the selected DE mRNAs and DE miRNAs through TargetScan (http://www.targetscan.org/vert_72/). TargetScan predicts biological targets of miRNAs by searching for the presence of conserved sites that match the seed region of each miRNA (Fromm et al., 2015). In brief, we not only searched for the presence of conserved sites among these DE mRNA that matched the seed region of DE miRNA but also searched for DE miRNA containing matched seed region for DE mRNA. These poorly conserved sites were excluded. Only these enrolled miRNA–mRNA pairs with opposite expression levels in EC samples as compared with normal tissues both presented in our dataset were adapted for further study.

### Cell Culture and Luciferase Reporter Assay

Cell culture and Luciferase reporter assay were very similar with those in our previous study (Zhou et al., 2012). Briefly, 293T cell and human endometrial cancer Ishikawa cell were cultured in Dulbecco’s modified Eagle’s medium (DMEM; Gibco, USA) supplemented with 10% fetal bovine serum (FBS; Gibco) and penicillin (100 U/ml). The 300-nt-long 3′-UTR (wild type or mutant) of EMX1 containing the predicted conserved binding sites for miR-497 was cloned into H306 pMIR-LUC vectors purchased from Obio Technology (Shanghai, China). Cells were plated at a density of 1 × 10^5^ in 12-well plate. After 24 h, the pMIR-LUC reporters were co-transfected with either miR-497 mimics or control using Lipofectamine 3000. Luciferase activity normalized to the Renilla Luciferase was measured by the Dual-Luciferase assay (Promega, USA) according to the manufacturer’s instructions after 48 h on the Luminometer (Promega, USA). The assay was repeated three times.

### RNA Extraction and Quantitative RT-PCR

Total RNA was extracted from cells with isolator reagent (Vazyme, China). After measurement of the RNA concentration, cDNAs were generated from reverse transcription with the HiScript II 1st Strand cDNA Synthesis Kit (Vazyme, China). The expression level of miR-497 was measured according to the instructions of the ChamQ^TM^ Universal SYBR qPCR Master Mix kit (Vazyme, China) using the ABI-7300 Real-Time PCR Detection System (Applied Biosystems, USA). The bulge-loop^TM^ miRNA Primer Sets (one RT primer and a pair of qPCR primers) specific for miR-497 were purchased from RiboBio (Guangzhou, China). The level of miR-497 was normalized to U6. Fold changes were calculated using the 2^−ΔΔCt^ method. Each plate was run in triplicate.

### Statistical Analysis

Logistic regression and *t* test were used to evaluate the relationship between gene expression and clinical–pathologic features. For gene expression level, the cutoff value was determined by its absolute median expression level (high- and low-expression groups). For the analysis of survival for patients with paired miRNA/mRNA, the median rate of miRNA/mRNA expression was used to divide groups for low and high rates. In logistic regression, the absolute gene expression level (high- and low-expression group) was used as categorical dependent variable, while clinical–pathologic feature was used as independent variable. Among all the clinical–pathologic features, age and BMI were calculated as continuous variables and the rest as categorical variables. Kaplan–Meier method and Cox regression were used to analyze clinicopathologic characteristics associated with OS. The influence of gene expression on survival along with other clinical characteristics was determined by multivariate Cox analysis. *P* value < 0.05 was set as the cutoff point. *P < 0.05, **P < 0.01, and ***P < 0.001.

## Results

### Identification of DE mRNAs and DE miRNAs With a Poorer Survival in EC

**Figure 1:**
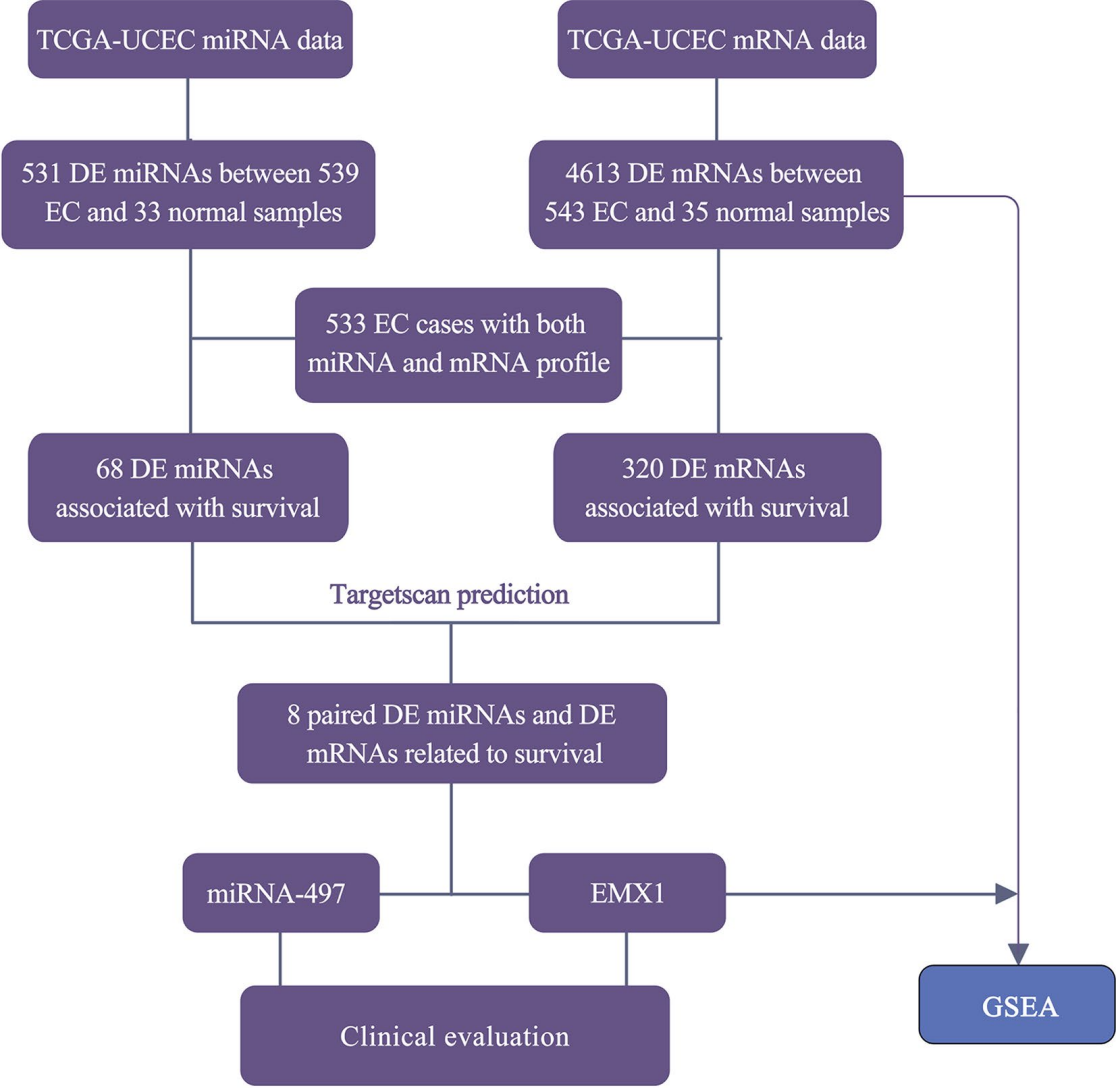
Workflow for the screening and evaluation of DE mRNAs and DE miRNAs between EC and normal samples.

The RNA-Seq profile data of 543 EC and 35 normal cases were downloaded from TCGA database along with the miRNA-seq data of 539 EC and 33 normal samples. Among them, 533 patients shared two expression profiles (**Figure 1**). Under the threshold of adj. *P* < 0.05 and fold-change value larger than 2, a total of 4,613 DE mRNAs (3,221 upregulated and 1,392 downregulated) and 531 DE miRNAs (374 upregulated and 157 downregulated) were identified in EC compared with normal samples. The heatmaps and volcano plots are shown in **Supplementary Figure 1**.

**Figure 2:**
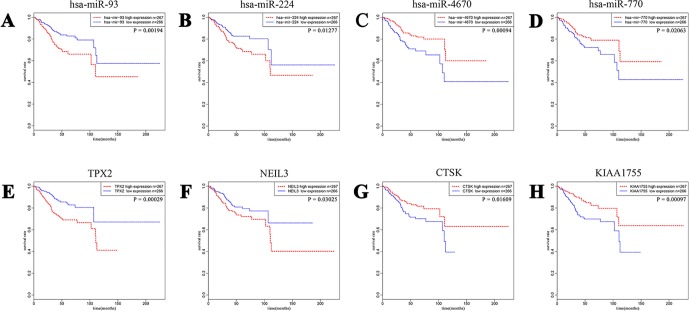
A total of 320 out of 4,613 DE mRNAs and 68 out of 531 DE miRNAs associated with a poorer survival rate were chosen. The eight plots show Kaplan–Meier curves of the partial DE miRNAs and DE mRNAs between cases of high-expression group and low-expression group divided according to the median expression level. **(A** and **B)** Upregulated miRNA-93 and miRNA-224. **(C** and **D)** Downregulated miRNA-4670 and miRNA-770. **(E** and **F)** Upregulated TPX2 and NEIL3. **(G** and **H)** Downregulated CTSK and KIAA1755.

To have a better understanding of the relationship between DEGs and patient survival, we conducted the “survival” package in R language to draw Kaplan–Meier curves according to the median expression level of DEGs among 533 patients. These upregulated and downregulated genes with a significantly poorer survival (*P* < 0.05), respectively, were determined. A total of 320 (280 upregulated and 40 downregulated) out of 4,613 DE mRNAs and 68 (43 upregulated and 25 downregulated) out of 531 DE miRNAs were chosen for further study (**Supplementary Table 1**). Kaplan–Meier curves of the partial genes between the high-expression group and low-expression group are shown in **Figure 2**.

### Functional and Pathway Enrichment Analysis

**Figure 3:**
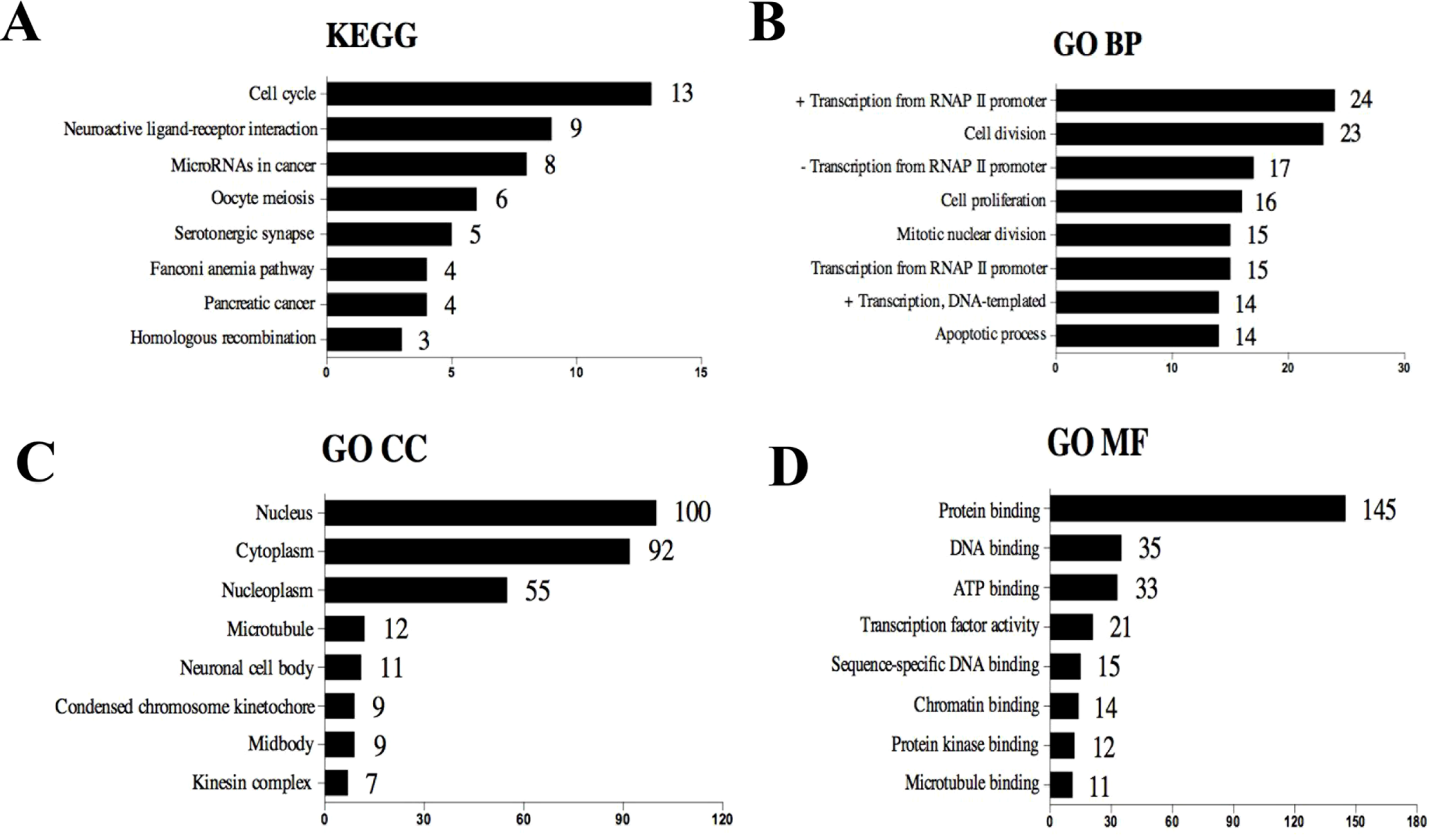
Top eight (sorted by count) KEGG pathways and GO terms identified among DEGs associated with survival using DAVID. **(A)** KEGG pathways. **(B)** GO term of Biological Process. **(C)** GO term of Cellular Component. **(D)** GO term of Molecular Function. “+” represents positive regulation, and “−” represents negative regulation.

The selected 320 DE mRNAs associated with survival were uploaded to DAVID to perform KEGG pathways and GO enrichment analysis. KEGG analysis revealed that these DEGs were mostly enriched in cell cycle, neuroactive ligand–receptor interaction, microRNAs in cancer, oocyte meiosis, and serotonergic synapse signaling pathways (**Figure 3A**). The top five enriched GO biological process (BP) terms included positive regulation of transcription from RNA polymerase II promoter, cell division, negative regulation of transcription from RNA polymerase II promoter, cell proliferation, and mitotic nuclear division. Nucleus, cytoplasm, nucleoplasm, microtubule, and neuronal cell body were the five most enriched GO terms for cellular component (CC). The top five enriched GO molecular function (MF) terms were protein binding, DNA binding, ATP binding, transcription factor activity, and sequence-specific DNA binding (**Figures 3B–D**). The most significantly enriched pathways and enrichment terms are also shown in **Supplementary Table 2**.

### Prediction Roles of Identified DE miRNAs and Their Potential Targeted mRNAs Associated With Survival

**Figure 4:**
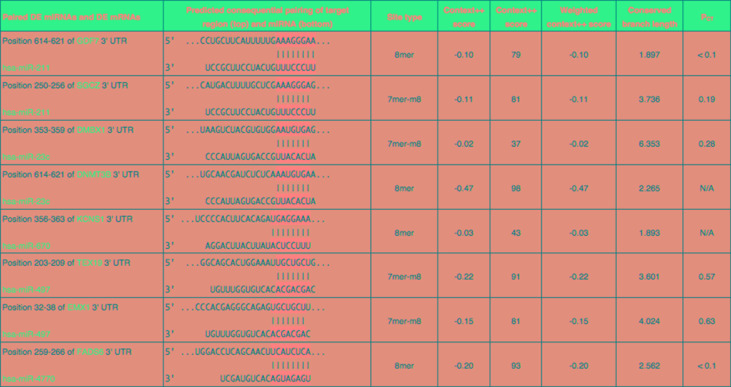
Eight predicted consequential pairings of DE miRNAs and target regions of DE mRNAs associated with survival. These enrolled miRNA–mRNA pairs not only contained conserved binding sites but also had opposite expression levels in EC samples as compared with normal tissues.

**Figure 5:**
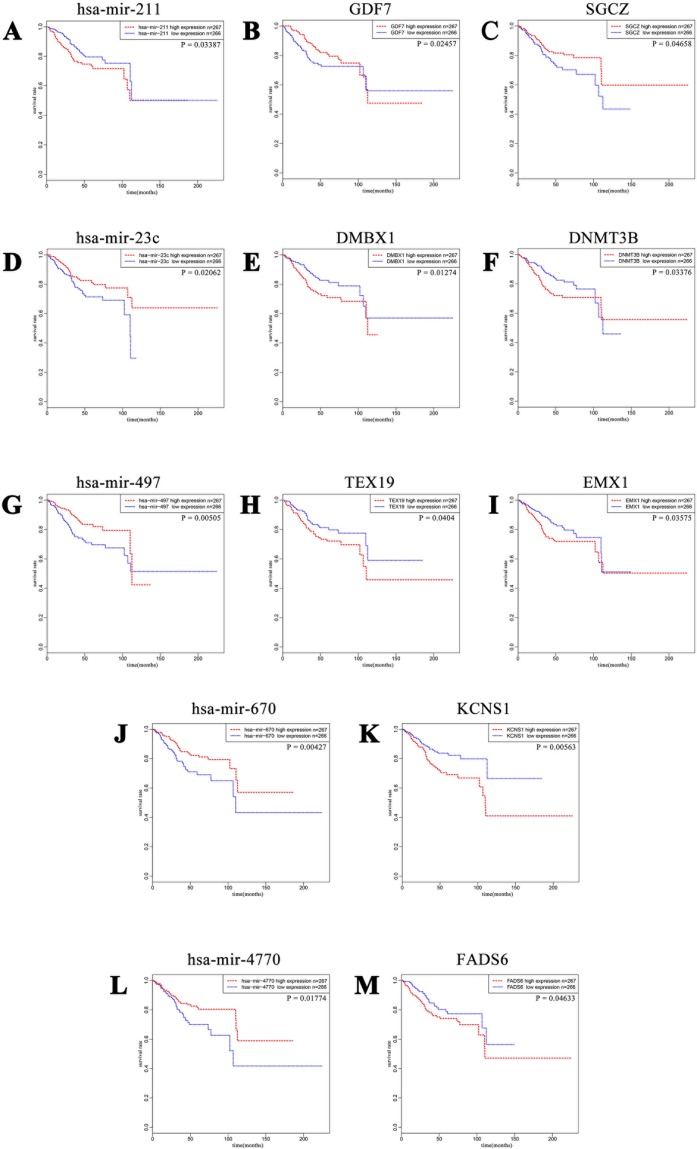
Kaplan–Meier curves of five DE miRNAs and eight potential target DE mRNAs, with one upregulated miRNA **(A)** miR-211 paired with two downregulated mRNAs **(B)** GDF7 and **(C)** SGCZ) and four downregulated miRNAs **(D)** miR-23c, **(G)** miR-497, **(J)** miR-670, and **(L)** miR-4770) paired with six upregulated mRNAs **(E)** DMBX1, **(F)** DNMT3B, **(H)** TEX19, **(I)** EMX1, **(K)** KCNS1, and **(M)** FADS6). For each gene, patients were divided into high-expression group and low-expression group according to the median expression level.

In order to better understand the role of DE miRNAs and their potential targeted DE mRNAs related to survival, we made target predictions through TargetScan. These genes containing conserved target sites, which matched the seed regions of miRNAs, were in our consideration. Besides, we only accepted these DE miRNAs and DE mRNAs with opposite expression levels in view of the silence effect of miRNA targeting. As a result, we predicted that 5 out of 68 DE miRNAs could interact with 8 out of 320 DE mRNAs (**Figure 4**), with one upregulated miRNA paired with two downregulated mRNAs and four downregulated miRNAs paired with six upregulated mRNAs (**Table 1**). The survival curves are also shown in **Figure 5**.

**Table 1:** DE miRNAs and their potential target DE mRNAs associated with survival.

	DE miRNA	*P* value*		DE mRNA	*P* value*
Upregulated			Downregulated		
	hsa-miR-211	0.03387		GDF7	0.02457
				SGCZ	0.04658
Downregulated			Upregulated		
	hsa-miR-670	0.00427		KCNS1	0.00563
	hsa-miR-4770	0.01774		FADS6	0.04633
	hsa-miR-23c	0.02062		DMBX1	0.01274
				DNMT3B	0.03376
	**hsa-miR-497**	**0.00505**		**EMX1**	**0.03575**
				TEX19	0.04040

*P value < 0.05 represented a significantly different OS rate between patients grouped according to the median level of gene expression.

The bolded miR-497/EMX1 was selected as a representative of eight miRNA/mRNA pairs for subsequent study.

**Figure 6:**
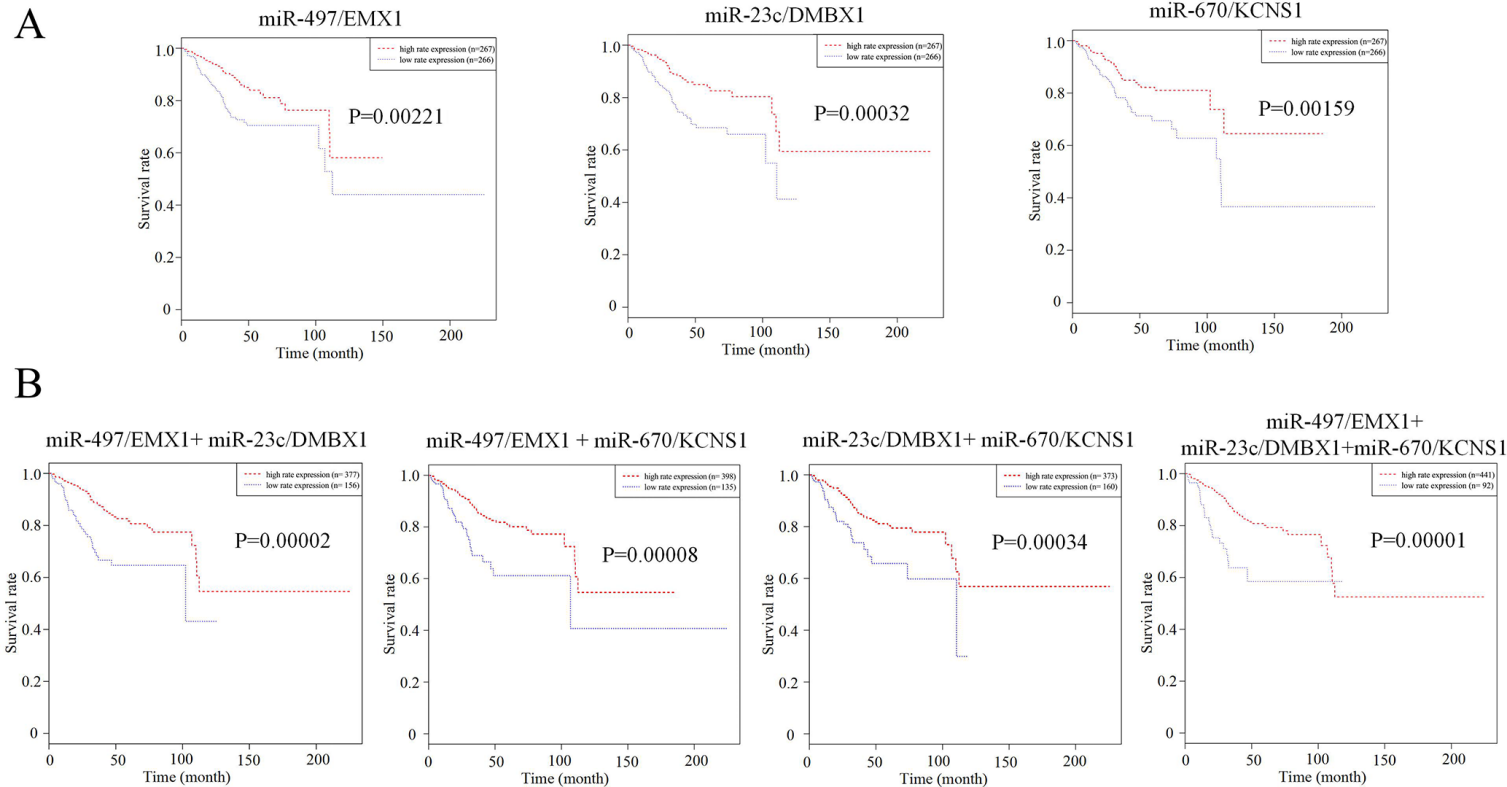
Prediction roles of identified DE miRNAs and their potential targeted mRNAs associated with survival. **(A)** Kaplan–Meier curves for patients with rate of three single paired miRNA/mRNA (miR-497/EMX1, miR-23c/DMBX1, and miR-670/KCNS1) expression. **(B)** Kaplan–Meier curves for patients with simultaneous presence of rates for more than one miRNA/mRNA pair among these three selected pairs. The median rate of miRNA/mRNA expression was used to divide groups into low and high rates.

We further tried to assess the differences between high and low rates of expression levels for the eight miRNA/mRNA pairs under the cutoff of median rate. As shown in **Figure 6A**, patients with low rate of three paired miRNA/mRNA (miR-497/EMX1, miR-23c/DMBX1, and miR-670/KCNS1) expression had a significantly poorer survival, while the rest seemed no different (data were not shown). Furthermore, under each cutoff of median rate, there were 156 patients carrying low rates of both miR-497/EMX1 and miR-23c/DMBX1 expression, 135 patients carrying low rates of both miR-497/EMX1 and miR-670/KCNS1 expression, 160 patients carrying low rates of both miR-670/KCNS1 and miR-23c/DMBX1 expression, and 92 patients carrying low rates of all the three miRNA/mRNA pairs (**Table 2**). Surprisingly, these patients with the simultaneous presence of these two or three low miRNA/mRNA pairs had a significantly poorer survival rate (**Figure 6B**). These results strongly verified the validity and prediction capacity of our analysis.

**Table 2:** Patients with the simultaneous presence of low rates of miRNA/mRNA pairs.

Low rate of miRNA/mRNA pairs	N	%	*P* value*
miR-497/EMX1 + miR-23c/DMBX1	156	29.27	0.00002
miR-497/EMX1 + miR-670/KCNS1	135	25.33	0.00008
miR-23c/DMBX1 + miR-670/KCNS1	160	30.02	0.00034
miR-497/EMX1 + miR-23c/DMBX1 + miR-670/KCNS1	92	17.26	0.00001

*P value represented a significantly different OS rate between patients with low and high rates of miRNA/mRNA expression.

### Clinical Evaluation of miR-497 and EMX1 Expression in EC

#### Patient Characteristics

We downloaded the clinical data of 533 primary EC from http://www.cbioportal.org. Patient characteristics are shown in **Table 3**. Median age at diagnosis of our study cohort was 64 year old (range 31–90 years old); 72.6% of the study group were white, with the rest scattered among other races. Most of the patients were endometrioid endometrial adenocarcinoma (EA; *n* = 402, 75.4%), 20.5% (*n* = 109) were serous, and 22 (4.1%) were mixed serous and endometrioid. The histopathologic distribution of EC was well differentiated (18.2%), moderately differentiated (22.5%), and poorly differentiated (59.3%). The cancer status included 419 cases without tumor (84.5%) and 77 cases with tumor (15.5%). In all patients, 32.7% had deep myometrial invasion, and 14.2% had positive peritoneal washings. About 16.8% of all patients had positive pelvic lymph nodes, while 10.3% had positive para-aortic lymph nodes. Stages I, II, III, and IV comprised 62.9%, 9.0%, 22.8%, and 5.3%, respectively. Median follow-up for subjects alive at last contact was 23.5 months (range 0–190 months).

**Table 3:** Clinical characteristics of TCGA EC patients.

Clinical characteristics		Total	%
Age at diagnosis (years)		64 (31–90)	
Race	White	363	72.6
	Black or African American	105	20.9
	Asian	20	3.9
	Other	13	2.6
BMI		504	
	Median BMI (range)	32.05 (17.36–81.64)	
Menopause status	Premenopausal	34	6.6
	Postmenopausal	438	86.6
	Peri-menopausal	17	3.4
	Indeterminate	17	3.4
Histologic grade	G1	97	18.2
	G2	120	22.5
	G3	316	59.3
Status	Tumor free	419	84.5
	With tumor	77	15.5
Histology	Serous endometrial adenocarcinoma	109	20.5
	Mixed serous and endometrioid	22	4.1
	Endometrioid endometrial adenocarcinoma	402	75.4
Stage	I	335	62.9
	II	48	9.0
	III	122	22.8
	IV	28	5.3
Myometrial invasion	Superficial (≤50%)	311	67.3
	Deep (>50%)	151	32.7
Peritoneal cytology	Negative	343	85.8
	Positive	57	14.2
Pelvic lymph nodes	Negative	361	83.2
	Positive	73	16.8
Para-aortic lymph nodes	Negative	322	89.7
	Positive	37	10.3

#### miR-497, EMX1 Expression, and Association With Clinicopathologic Variables

**Figure 7:**
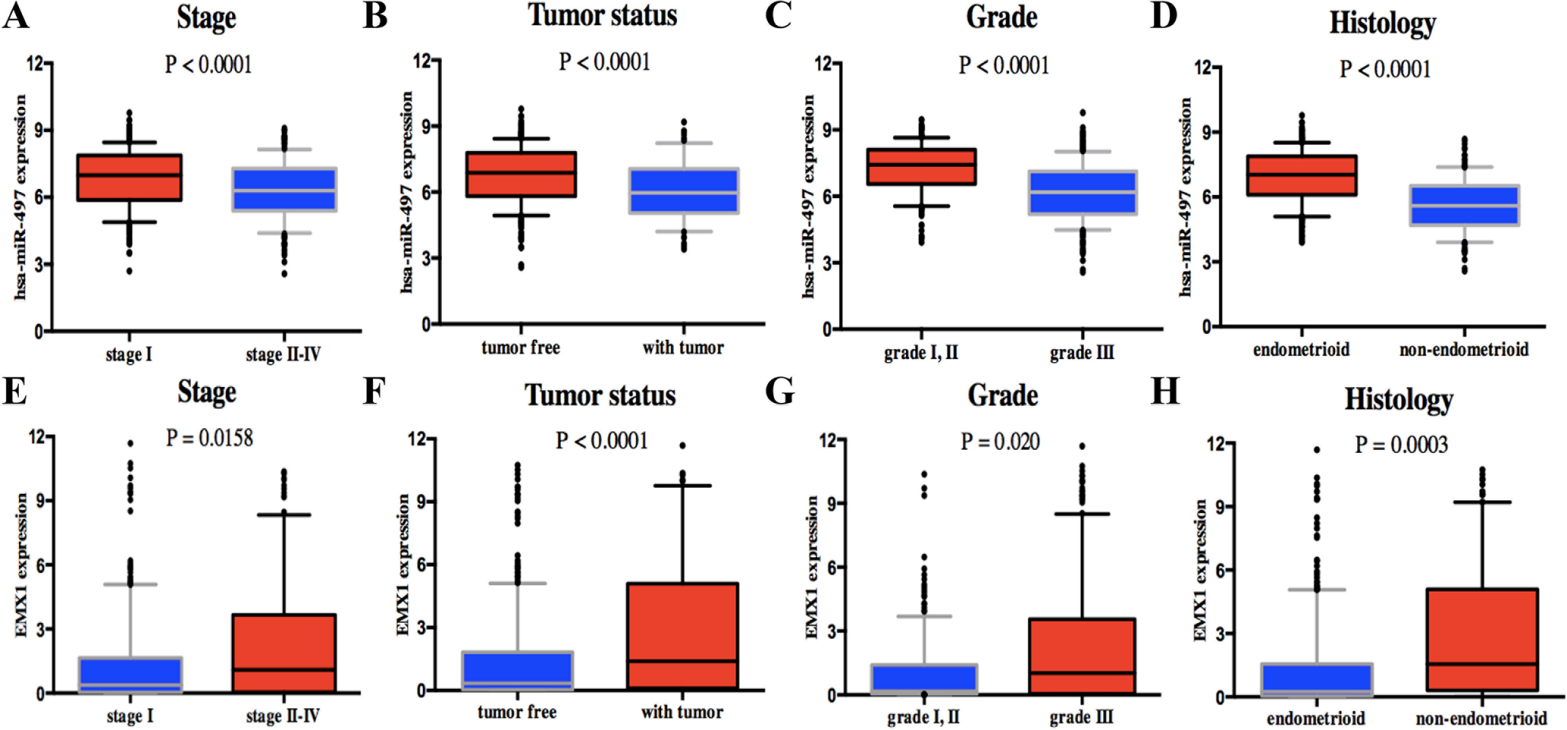
Association with DEG expression and clinicopathologic characteristics, including has-miR-497 expression associated with **(A)** stage, **(B)** tumor status, **(C)** grade, and **(D)** histology and EMX1 expression associated with **(E)** stage, **(F)** tumor status, **(G)** grade, and **(H)** histology. *P* values were calculated using *t* test.

Univariate analysis using logistic regression revealed that miR-497 expression as a categorical dependent variable (based on median expression value) was associated with adverse prognostic clinical pathological features (**Table 4**). Decreased miR-497 expression in EC was significantly associated with high grade (OR = 0.228 for G1, G2 vs. G3), stage (OR = 0.470 for I vs. II, III, and IV), histology (OR = 0.175 for EA vs. non-EA), tumor status (OR = 0.391 for tumor free vs. with tumor), pelvic lymph node metastasis (OR = 0.294 for positive vs. negative), para-aortic lymph node metastasis (OR = 0.232 for positive vs. negative) and peritoneal washings (OR = 0.510 for positive vs. negative) (all *P* values < 0.05, **Figures 7A–D**).

**Table 4:** Univariate miR-497 expression* association with clinicopathologic characteristics (logistic regression).

Clinicopathologic variable	Total (*N*)	Odds ratio	*P* value
Age (continuous)	531	0.971 (0.955–0.986)	0.080
Race (white vs. other)	501	0.471 (0.315–0.704)	0.000
BMI (continuous)	503	1.039 (1.018–1.061)	0.000
Grade (G1, G2 vs. G3)	533	0.228 (0.157–0.331)	0.000
Status (tumor free vs. with tumor)	496	0.391 (0.234–0.654)	0.000
Histology (EA vs. non-EA)	533	0.175 (0.110–0.280)	0.000
Stage (I vs. II, III, IV)	533	0.470 (0.328–0.672)	0.000
Myometrial invasion (deep vs. superficial)	462	0.736 (0.498–1.087)	0.123
Peritoneal cytology (positive vs. negative)	400	0.510 (0.286–0.910)	0.023
Pelvic lymph nodes (positive vs. negative)	434	0.294 (0.169–0.512)	0.000
Para-aortic lymph nodes (positive vs. negative)	359	0.232 (0.103–0.522)	0.000

*Categorical dependent variable, greater or less than the median expression level.

On the contrary, high EMX1 expression was associated with poor prognostic clinicopathologic characteristics (**Table 5**). Overexpressed EMX1 in EC was associated with clinically advanced stage (OR = 1.668 for I vs. II, III, and IV), high grade (OR = 2.325 for G1, G2 vs. G3), histology (OR = 3.060 for EA vs. non-EA), tumor status (OR = 2.342 for tumor free vs. with tumor), and para-aortic lymph node metastasis (OR = 2.547 for positive vs. negative) (all *P* values < 0.05, **Figures 7E–H**).

**Table 5:** Univariate EMX1 expression* association with clinicopathologic characteristics (logistic regression).

Clinicopathologic variable	Total (*N*)	Odds ratio	*P* value
Age (continuous)	531	1.021 (1.006−1.037)	0.143
Race (white vs. other)	501	1.476 (0.995−2.190)	0.053
BMI (continuous)	503	0.966 (0.947−0.986)	0.001
Grade (G1, G2 vs. G3)	533	2.325 (1.632−3.313)	0.000
Status (tumor free vs. with tumor)	496	2.342 (1.406−3.899)	0.001
Histology (EA vs. non-EA)	533	3.060 (2.004−4.672)	0.000
Stage (I vs. II, III, IV)	533	1.668 (1.170−2.379)	0.005
Myometrial invasion (deep vs. superficial)	462	1.377 (0.932−2.034)	0.108
Peritoneal cytology (positive vs. negative)	400	1.365 (0.776−2.400)	0.280
Pelvic lymph nodes (positive vs. negative)	434	1.456 (0.876−2.420)	0.147
Para-aortic lymph nodes (positive vs. negative)	359	2.547 (1.217−5.327)	0.013

*Categorical dependent variable, greater or less than the median expression level.

These results suggested that EC patients with low expression of miR-497 and high expression of EMX1 were prone to progress to a more advanced stage than those with high expression of miR-497 and low expression of EMX1.

#### Survival Outcomes and Multivariate Analysis

Kaplan–Meier survival analysis showed that EC with low expression of miR-497 had a worse prognosis than that with high expression (**Figure 5G**). The univariate analysis revealed that reduced miR-497 correlated significantly with a poor OS (HR: 0.536; 95% CI: 0.345–0.831; *P* = 0.005).

Meanwhile, Kaplan–Meier survival analysis suggested that patients with high EMX1 expression also had a poorer prognosis than those with low EMX1 expression (**Figure 5I**). And, univariate analysis revealed that high EMX1 expression correlated significantly with a poorer OS rate (HR: 1.563, 95% CI: 1.019–2.396; *P* = 0.041).

Other clinicopathologic factors associated with poor survival include high grade, histology, advanced stage, myometrial invasion, peritoneal washings, and lymph node metastasis (**Table 6A**).

**Table 6:** **a**. Overall survival and associations with clinicopathologic characteristics using Cox regression. **b.** Multivariate survival model after variable selection.

Clinicopathologic variable	Hazard ratio (95% CI)	*P* value
a.		
Age (continuous)	1.035 (1.015−1.056)	0.001
hsa-miR-497 expression (low vs. high)	0.536 (0.345–0.831)	0.005
EMX1 expression (high vs. low)	1.563 (1.019–2.396)	0.041
Race (white vs. other)	0.944 (0.585–1.521)	0.812
BMI (continuous)	0.989 (0.965–1.013)	0.360
Grade (G1, G2 vs. G3)	3.636 (2.084–6.342)	0.000
Status (tumor free vs. with tumor)	8.553 (5.524–13.242)	0.000
Histology (EA vs. non-EA)	2.944 (1.930–4.492)	0.000
Stage (I vs. II, III, IV)	3.775 (2.429–5.866)	0.000
Myometrial invasion (deep vs. superficial)	3.976 (2.490–6.347)	0.000
Peritoneal cytology (positive vs. negative)	4.842 (2.937–7.982)	0.000
Pelvic lymph nodes (positive vs. negative)	4.672 (2.841–7.682)	0.000
Para-aortic lymph nodes (positive vs. negative)	3.906 (2.083–7.325)	0.000
b.		
hsa-miR-497 expression (low vs. high)	0.587 (0.363–0.951)	0.030
EMX1 expression (high vs. low)	1.479 (0.929–2.353)	0.099
Myometrial invasion (deep vs. superficial)	3.618 (2.260–5.792)	0.000

In multivariate analysis (**Table 6b**), miR-497 remained an independent prognostic variable for OS, with an HR of 0.587 (CI: 0.363–0.951, *P* = 0.030), along with myometrial invasion.

### EMX1 Was a Direct Target of miR-497

**Figure 8:**
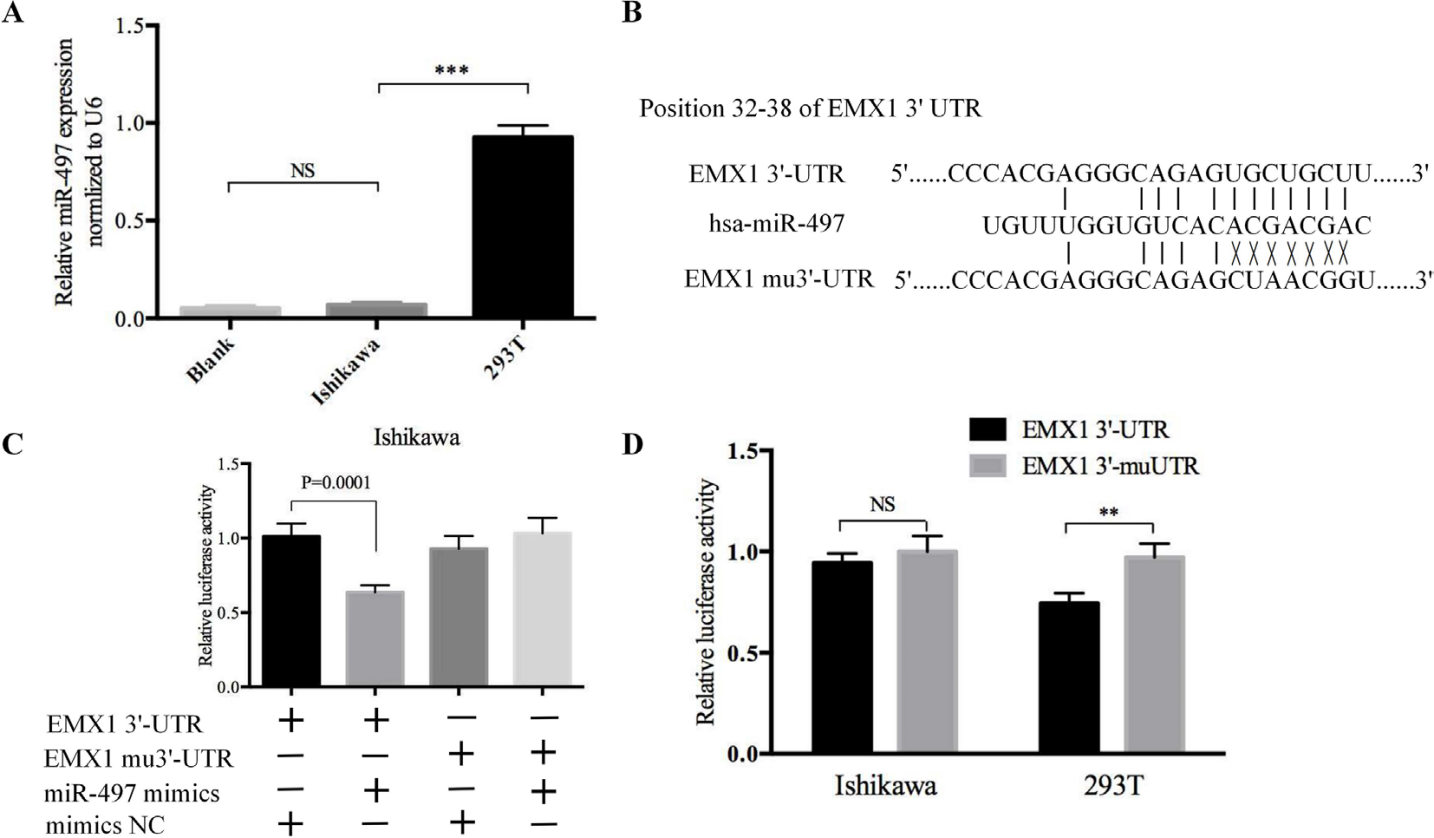
The luciferase reporter assay confirmed that EMX1 was a direct target of miR-497. **(A)** The expression levels of miR-497 in Ishikawa and 293T cells. **(B)** The seed regions of miR-497 of EMX1 3′-UTR and mu3′-UTR. **(C)** The luciferase activity of Ishikawa cells co-transfected with miR-497 mimics or mimics NC, and pMIR-LUC-EMX1-3′-UTR (EMX1 3′-UTR) or pMIR-LUC-EMX1-mu3′-UTR (EMX1 mu3′-UTR). **(D)** The luciferase activity of Ishikawa and 293T cells transfected with EMX1 3′-UTR or EMX1 mu3′-UTR. **P < 0.01, ***P < 0.001.

To explore whether EMX1 was a direct target of miR-497, we first detected the miR-497 levels in 293T and Ishikawa cells. As shown in **Figure 8A**, the miR-497 level in Ishikawa cell was very low, while it was much higher in 293T cell. We next performed a Dual-Luciferase reporter assay. The binding sites for miR-497 with EMX1 wild-type 3′-UTR (EMX1 3′-UTR) and EMX1 mutant 3′-UTR (EMX1 mu3′-UTR) are shown in **Figure 8B**. There was no loss of luciferase activity in Ishikawa cells with co-transfection of miR-497 mimics and mutated 3′-UTR of EMX1 plasmid, while a significant luciferase activity decrease was observed in cells co-transfected with miR-497 mimics and EMX1 3′-UTR plasmid (**Figure 8C**, *** *P* = 0.0001). We also performed the luciferase assay in 293T cells, which showed an increased relative luciferase activity of the construct having the mutated binding site compared with that of the wild-type site (**Figure 8D**, ** *P* < 0.01). These data indicated that EMX1 was a direct target of miR-497.

### GSEA Identified EMX1-Related Oncogenic Signaling Pathways

The GSEA method is used to deal with continuous data and identify gene sets that are enriched at the top (overexpressed vs. control) or bottom (underexpressed) of a ranked gene list (Subramanian et al., 2005). We performed GSEA with the ordered list of preliminary screening DEGs according to their correlation with EMX1 expression. MSigDB Collection (c6.all.v6.2.symbols.gmt), which represents signatures of cellular pathways often dysregulated in cancer, was applied to our GSEA analysis at the phenotype of EMX1 expression level. Twenty-one signaling pathways were significantly enriched based on their NES (NOM *P* value < 0.05, FDR *q*-value < 0.25, **Table 7**). **Supplementary Figure 2** shows that part of those oncogenic gene-involved cellular pathways, including PIGF, SRC, JNK KRAS.AMP.LUNG, PTEN, and E2F3, were differentially enriched in EMX1 high-expression phenotype.

**Table 7:** Gene Set Enrichment Analysis in EMX1 phenotype.

MSigDB collection	Gene set name	NES	NOM *P* val	FDR *q*-val
c6.all.v6.2.symbols.gmt	PIGF_UP.V1_DN	1.808	0.0027	0.2133
	SRC_UP.V1_UP	1.756	0.0071	0.1653
	JNK_DN.V1_DN	1.661	0.0107	0.2138
	KRAS.AMP.LUNG_UP.V1_DN	1.739	0.0116	0.1629
	PTEN_DN.V2_DN	1.648	0.0175	0.1952
	E2F3_UP.V1_UP	1.671	0.0217	0.2194
	KRAS.LUNG_UP.V1_DN	1.806	0.0218	0.1624
	KRAS.50_UP.V1_DN	1.799	0.0235	0.1374
	ATM_DN.V1_UP	1.628	0.0236	0.1827
	PRC2_EZH2_UP.V1_DN	1.620	0.0245	0.1810
	NOTCH_DN.V1_UP	1.560	0.0265	0.2069
	PRC2_SUZ12_UP.V1_DN	1.539	0.0288	0.1912
	RB_P107_DN.V1_UP	1.632	0.0339	0.2043
	P53_DN.V2_UP	1.628	0.0356	0.1952
	NOTCH_DN.V1_DN	1.547	0.0372	0.1962
	ATF2_UP.V1_UP	1.579	0.0396	0.2249
	KRAS.KIDNEY_UP.V1_DN	1.557	0.0396	0.2005
	PKCA_DN.V1_UP	1.484	0.0417	0.2356
	JAK2_DN.V1_UP	1.568	0.0435	0.2168
	CAHOY_NEURONAL	1.674	0.0464	0.2415
	RB_P130_DN.V1_UP	1.574	0.0490	0.2190

NES, normalized enrichment score; NOM, nominal; FDR, false discovery rate. Gene sets with NOM P val < 0.05 and FDR q-val < 0.25 are considered significant.

## Discussion

In the recent years, more and more evidence has revealed that miRNAs play important roles in the development and progression of tumors. They may serve as molecular indicators of prognosis and targets for oncotherapy (Jonas and Izaurralde, 2015). In endometrial cancer, a number of studies have looked at the miRNA profiles using tumor tissues or blood samples with a goal to identify disease-specific biomarkers (Srivastava et al., 2017). However, previous studies usually focused on one or two specific miRNAs and their target genes based on their potential to regulate diverse biological processes. Few researchers carried out studies on the relationship between whole miRNA profile and their potential target mRNAs based on their impact on patient survival.

A miRNA-based research data source of which was also a TCGA-UCEC project was carried out not long ago (Wang et al., 2018). It identified miRNAs that were correlated with the occurrence and progression of EC and established a six-miRNA (miR-15a. MIMAT0000068, miR-142. MIMAT0000433, miR-142. MIMAT0000434, miR-3170. MIMAT0015045, miR-1976. MIMAT0009451, and miR-146a. MIMAT0000449) expression signature as a predictor for the OS of patients with EC. In this study, the six-miRNA model was based on these 144 DE miRNAs resulting only from 15 patients with corresponding miRNA expression of the paired adjacent tissues. Another miRNA-based study on TCGA-UCEC project focused on the difference of miRNA profile between metastatic and nonmetastatic ECs (Zhu et al., 2018). It revealed that four miRNAs (miR-1247 miR-3200, miR-150, and miR-301b) were differently expressed between two groups, miR-1247 was associated with metastasis of EC to the lung, and miR-3200 is associated with the clinical stage of EC. The researchers also performed functional enrichment analysis with these predicted potential target genes of the four miRNAs, which showed that they might be involved in multiple pathways of cancer, including the Wnt, NOTCH, and TGF-β signaling pathways and signaling pathways regulating pluripotency of stem cells. However, miRNAs can target multiple genes, and a single gene can be targeted by multiple miRNAs (Ambros, 2004). Unilateral research is not enough to illustrate the problem.

In this study, for the first time, we performed Kaplan–Meier analysis for all the DE mRNAs and DE miRNAs in a larger number of EC cases from TCGA project with the help of bioinformation technology. As a result, 320 out of 4,613 DE mRNAs and 68 out of 531 DE miRNAs with a significantly poorer survival were determined. For the selected DE mRNAs associated with survival, KEGG pathway and GO enrichment analysis revealed that these selected DE mRNAs were significantly enriched in several functional pathways, including microRNAs in cancer. We further made target predictions through TargetScan and found that 5 out of 68 DE miRNAs could interact with eight DE mRNAs from 320 DE mRNAs. However, since the cutoff value for dividing groups (high expression and low expression) was based on the absolute median, most *P* values for different OS rates of the selected paired genes were of borderline statistical significance. Xie et al. (2018) used median mRNA expression level of ALKBH1 as a separation for high- and low-expression groups based on another TCGA project of glioblastoma (*n* = 488), which also resulted in a minor difference (*P* = 0.0386) and provided evidence for ALKBH1 to be a potential therapeutically targetable node. Another way to perform analysis was by using the computing cutoff expression level for the best separation with the smallest *P* value on survival and different numbers in two groups for each gene, which was also our initial design and resulted in a much larger number of DE mRNAs and miRNAs related to survival. To narrow the results of genes associated with survival, we finally chose the median truncation value, though some genes with potential predictability may be lost in our study.

Since each group of patients with a single miRNA or mRNA was made independently and their composition might be different, it would be interesting to determine differential expression of more than one miRNA or mRNA simultaneously in the patients. We tried to assess the differences between high and low rates of expression levels for the eight miRNA/mRNA pairs. As a result, patients with a low rate of three paired miRNA/mRNA (miR-497/EMX1, miR-23c/DMBX1, and miR-670/KCNS1) had a significantly poorer survival, while the rest seemed no different. As the presence of a single miRNA/mRNA pair is clearly not sufficient to predict the outcome of a patient having EC, we further verified the simultaneous presence of low rate of more than one miRNA/mRNA pair among these three selected pairs in the same group of patients. Surprisingly, the simultaneous presence of these selected low miRNA/mRNA pairs occurred in most EC patients and resulted in a significantly poorer survival rate, which strongly verified the validity and prediction capacity of our analysis. The experimental luciferase reporter assay confirmed that EMX1 was a direct target of miR-497. Clinical evaluation was assessed on miR-497 and EMX1 expression levels. Interestingly, they had an opposite significant relationship with several clinicopathologic characteristics besides survival. Multivariate analysis also demonstrated that miR-497 remained independent prognostic variables for OS. These data also provided evidence for the value of our prediction analysis in EC.

Among 68 DE miRNAs related to a poorer survival, 43 miRNAs were upregulated and 25 were downregulated. In 2013, Torres et al. conducted a study that aimed to reveal the relationship between DE miRNAs and EC (Torres et al., 2013). They found that upregulated miR-1228 was significantly associated with a poorer survival, which was consistent with our research result. Another research revealed that lower expression of miR-101, miR-10b, miR-139-5p, miR-152, miR-29b, and miR-455-5p was significantly correlated with poor OS and that decreased expression of miR-152 was a statistically independent risk factor for OS in EC (Hiroki et al., 2010). Mitamura et al. found that miR-31 was significantly upregulated in the EC patients with a high risk of recurrence compared with that observed in the low-risk patients, and this higher expression correlated with a poorer progression-free survival (Mitamura et al., 2014).

miRNAs mainly function and result in silence effect at posttranscriptional level by base pairing with the 3′-UTR of their target mRNAs completely or incompletely (Moran et al., 2017). In the present study, we predicted eight potential target DE mRNAs for the five DE miRNAs, including miR-211, miR-23c, miR-670, miR-497, and miR-4770. To our knowledge, there is no basic experimental evidence for the eight pairs of miRNAs and mRNAs predicted. One of the mostly studied miRNAs, miR-497, was recognized as a tumor suppressor in many cancers (Zhao et al., 2013; Du et al., 2015; Zhao et al., 2015; Yang et al., 2016), which was also consistent with our result in EC. During our manuscript preparation, a meta-analysis (Feng et al., 2018) on the prognostic role of miR-497 in different cancer patients revealed that high-expression levels of miR-497 are less possible to have lymph node metastasis and have better overall survival, which indicated that miR-497 might be a potential biomarker and could be used to predict the better prognosis of different cancer types. A recent study showed that miR-497 negatively regulated glioma cells by targeting oncogene Wnt3α and that reduced expression of miR-497 was associated with poor disease-free and overall survival rates (Lu et al., 2018). It could also suppress clear cell renal cell carcinoma by targeting PD-L1, which was an immune-related oncogene (Qu et al., 2018). miR-497 could target SERPINE-1 and induce reversion of epithelial-to-mesenchymal transition in cutaneous squamous cell carcinoma (Mizrahi et al., 2018). However, these targets of miR-497 did not appear as a DE mRNA associated with survival in the present study. EMX1, a potential target of miR-497 predicted through TargetScan, also had a very close relationship with EC clinicopathologies and patient survival, which was in contrast to the miR-497 in our study. Asada et al. revealed that a high quartile of EMX1 methylation level had a significant univariate HR and a multivariate-adjusted HR of developing authentic metachronous gastric cancers (Asada et al., 2015). Its methylation level was also differently expressed in hepatocellular carcinoma (HCC), which showed a potential role in the development of HCC (Sun et al., 2018). However, most of the research on EMX1 was focused on brain development (Lim et al., 2015; Kobeissy et al., 2016). The relationship between miR-497/EMX1 and cancer needs further study.

Testis-expressed 19 (TEX19) was another potential target gene of miR-497 in our study. Zhong. et al. revealed that TEX19 exhibited increased expression in high-grade tumors and might represent a novel cancer-testis gene related to the progression of bladder cancer (Zhong et al., 2016). TEX19 was also required to drive cell proliferation in a range of cancer cell types, and its expression was linked to a poor prognosis for breast cancer, kidney cancer, prostate cancer, and glioma cancer (Planells-Palop et al., 2017). However, further study on its role in EC is needed.

Bu Y. et al. identified that miR-211 directly targets to Bmal1 and Clock in Burkitt’s lymphoma, thereby suppressing both circadian oscillation and ongoing protein synthesis to facilitate tumor progression (Bu et al., 2018). miR-23c, as a target of lncRNA MALAT1, could directly repress its target ELAVL1 and inhibit hyperglycemia-induced cell pyroptosis (Li et al., 2017). Shi C. et al. showed evidence that miR-670 could induce cell proliferation in hepatocellular carcinoma by targeting PROX1 (Shi and Xu, 2016), while our study predicted that it might play an important role in EC progression *via* targeting Potassium Voltage-Gated Channel Modifier Subfamily S Member 1 (KCNS1), which was also a DE mRNA associated with survival in our study. Previous studies on KCNS1 mainly focused on pain (Tsantoulas et al., 2018; Wonkam et al., 2018). Besides the downregulated expression level of KCNS1 in metastatic breast carcinoma (Savci-Heijink et al., 2016), little research has been carried out on the relationship between KCNS1 and cancer.

DNA methyltransferase 3B (DNMT3B), one of the eight paired genes in our study, was a methyltransferase responsible for *de novo* DNA methylation. A previous study also showed that it was significantly upregulated in both grade I and grade III ECs as compared with normal controls (Jin et al., 2005). DNMT3B-involved divergent DNA methylation pathways and protein synthesis required for posttranscriptional regulation may be implicated in the development of type I and type II ECs (Xiong et al., 2005a; Xiong et al., 2005b). However, little is known about the relationships between EC and the rest of the potential miRNA-targeted DE mRNAs, including growth differentiation factor 7 (GDF7), sarcoglycan zeta (SGCZ), diencephalon/mesencephalon homeobox 1 (DMBX1), and fatty acid desaturase 6 (FADS6). Further research is also needed.

There were emerging researches on EC using TCGA data. Studies revealed that L1CAM (Dellinger et al., 2016) and TFAP2B (Wu and Zhang, 2018) were the two DEGs associated with advanced clinicopathologic characteristics and independent predictors for survival in EC based on TCGA-UCEC data. However, the massive narrowing down process for the DEG screening was not shown. Another study focused on the mutation-expression profile and looked for guidance for EC drug discovery (Wong et al., 2016). Thus, while these studies and ours were all based on TCGA data for EC, the different focus and data analysis approaches led to the discovery of different aspects of the disease.

In this study, we identified a series of DE mRNAs and DE miRNAs associated with survival in EC. Furthermore, we predicted eight pairs of DE miRNAs and their potential target DE mRNAs related to survival. Clinical evaluation of downregulated miR-497 and paired upregulated EMX1 confirmed the value of our prediction analysis in EC. The simultaneous presence of low rate of these selected low miRNA/mRNA pairs (miR-497/EMX1, miR-23c/DMBX1, and miR-670/KCNS1) might have a better prediction value. Therefore, further studies are required to validate these findings.

## Author Contributions

XX, JW, and HZ designed the study, checked the data, and prepared the manuscript. TL, YW, JF, and QY performed data collection and statistical analysis. TL searched the literature and took part in the manuscript preparation. JW and HZ conceived and supervised the project.

## Funding

Funding was provided by the Natural Science Foundation of Jiangsu Province (BK20151096) and the Key Projects of National Health and Family Planning Commission of Nanjing City (ZKX17015) to HZ and the Fundamental Research Funds for the Central Universities (3332018179) to JW.

## Conflict of Interest Statement

The authors declare that the research was conducted in the absence of any commercial or financial relationships that could be construed as a potential conflict of interest.
