# Supplementary material for: miRNA–mRNA Associated With Survival in Endometrial Cancer
Source: Front Genet. 2019 Aug 20;10:743. doi: 10.3389/fgene.2019.00743 (PMC6710979; doi:10.3389/fgene.2019.00743)
Supplement: Supplementary file 1 [file Table_1.docx]

Supplementary Material

miRNA-mRNA associated with survival in endometrial cancer

Xiaofeng Xu^1#^, Tao Liu^1,2#^, Yijin Wang^1,3^, Jian Fu^4^, Qian Yang^5^, Jun Wu^1*^, and Huaijun Zhou^1*^

^1^Department of Gynecology, The Affiliated Drum Tower Hospital of Nanjing University Medical School, Nanjing, 210008, People's Republic of China

^2^Medical College, Nanjing University, Nanjing, 210008, People's Republic of China

^3^Medical College, Southeast University , Nanjing, 210008, People's Republic of China

^4^Department of Gynecology, Suqian People's Hospital of Nanjing Drum Tower Hospital Group, Suqian, 223800, People's Republic of China.

^5^Department of Gynecology and Obstetrics, The pukou Hospital of Nanjing, The Fourth Affiliated Hospital of Nanjing Medical University, Nanjing, 210031, People's Republic of China.

^#^ These authors contributed equally to this work.

* Correspondence: Dr. Huaijun Zhou: E-mail: [zhouhj2007@126.com](mailto:zhouhj2007@126.com) and Jun Wu, E-mail: [iamwujun2008@163.com](mailto:iamwujun2008@163.com), Department of Gynecology, Nanjing Drum Tower Hospital, 321 Zhongshan Road, Nanjing 210008, People's Republic of China

# 1 Supplementary Figures

**Supplementary Fig. 1.** Identification of 4613 DE mRNAs and 531 DE miRNAs in EC compared with normal samples. Heatmap (A) and Volcano plot (B) of the 3221 upregulated and 1392 downregulated DE mRNAs. Heatmap (C) and Volcano plot (D) of the 374 upregulated and 157 downregulated DE miRNAs. The red color represents high expression, and the green color represents low expression.


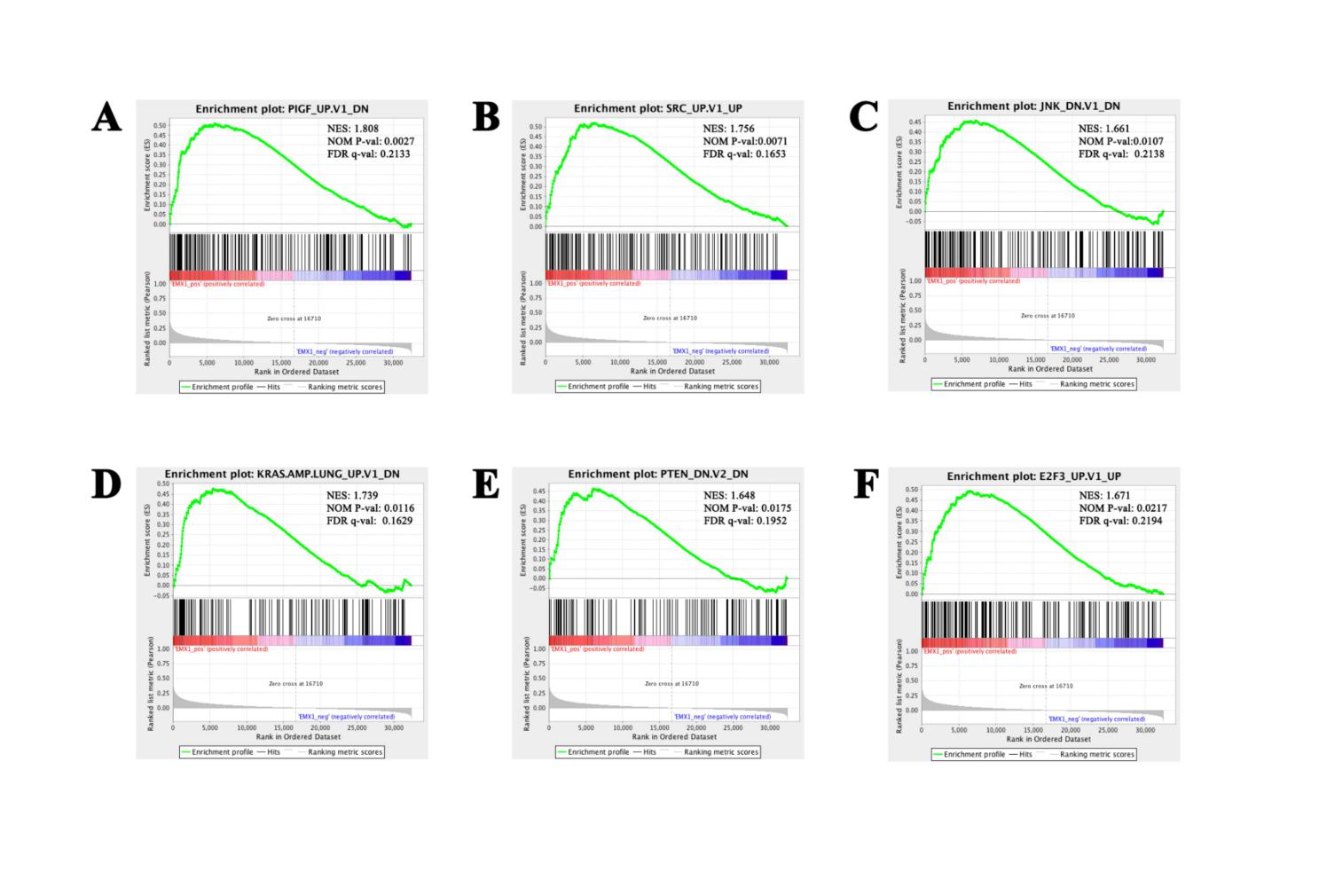


**Supplementary Fig. 2.** Gene sets enrichment analysis (GSEA) in EMX1 phenotype. The six plots show that partial of those oncogenic gene-involved cellular pathways, including PIGF (A), SRC (B), JNK (C), KRAS.AMP.LUNG (D), PTEN (E) and E2F3 (F) were differently enriched in EMX1 high expression phenotype.

**2 Supplementary Tables**

**Supplementary Table 1.** These 320 ( 280 upregulated and 40 downregulated ) DE mRNAs and 68 (43 upregulated and 25 downregulated ) DE miRNAs with a significant poorer survival were determined.

| Upregulated  mRNA | P Value* | Downregulated  mRNA | P Value* | Upregulated  miRNA | P Value* | Downregulated  miRNA | P Value* |
| --- | --- | --- | --- | --- | --- | --- | --- |
| ABCA4 | 0.04502 | ATP8B4 | 0.00391 | hsa-mir-1228 | 0.0487 | hsa-mir-1245a | 0.04297 |
| ACBD7 | 0.0420 | C1QTNF7 | 0.00015 | hsa-mir-1229 | 0.0034 | hsa-mir-1298 | 0.02125 |
| ACTL8 | 0.00023 | CACNA1H | 0.03847 | hsa-mir-1276 | 0.04175 | hsa-mir-23c | 0.02062 |
| ALG1L3P | 0.04068 | CBLN4 | 0.03247 | hsa-mir-1301 | 0.00641 | hsa-mir-2682 | 0.01145 |
| ALG1L5P | 0.00086 | CFD | 0.02456 | hsa-mir-138-2 | 0.02330 | hsa-mir-28 | 0.03624 |
| ALK | 0.00011 | CLEC9A | 0.01501 | hsa-mir-18a | 0.00160 | hsa-mir-3132 | 0.00469 |
| ANKLE1 | 0.01455 | CLIC2 | 0.04254 | hsa-mir-211 | 0.03387 | hsa-mir-3202-1 | 0.00139 |
| ANKRD34B | 0.01088 | CPED1 | 0.00983 | hsa-mir-216a | 0.03365 | hsa-mir-3202-2 | 0.00130 |
| ANLN | 0.00738 | CTSK | 0.01609 | hsa-mir-224 | 0.01277 | hsa-mir-3610 | 0.00960 |
| APLP1 | 0.00439 | DNASE1L3 | 0.01837 | hsa-mir-31 | 0.00002 | hsa-mir-4284 | 0.00872 |
| ARL14 | 0.02738 | ECM2 | 0.04251 | hsa-mir-3127 | 0.00972 | hsa-mir-4424 | 0.00370 |
| ASPM | 0.00106 | ECT2L | 0.00817 | hsa-mir-3200 | 0.01959 | hsa-mir-4524a | 0.00179 |
| ATP13A5 | 0.03452 | FAM13C | 0.0003 | hsa-mir-324 | 0.01352 | hsa-mir-4529 | 0.01208 |
| AUNIP | 0.04214 | FOXL2 | 0.03808 | hsa-mir-330 | 0.00597 | hsa-mir-4536-1 | 0.03162 |
| AURKA | 0.0009 | GDF7 | 0.02457 | hsa-mir-3680-1 | 0.04858 | hsa-mir-4670 | 0.00094 |
| B3GNT3 | 0.04536 | GIMAP8 | 0.04742 | hsa-mir-3691 | 0.01478 | hsa-mir-4705 | 0.00595 |
| B4GALNT2 | 0.03325 | GNA14 | 0.02089 | hsa-mir-3944 | 0.02296 | hsa-mir-4740 | 0.00053 |
| BARX1 | 0.00014 | GNG11 | 0.04874 | hsa-mir-421 | 0.02244 | hsa-mir-4770 | 0.01774 |
| BCRP2 | 0.02797 | GYPC | 0.04346 | hsa-mir-425 | 0.04465 | hsa-mir-497 | 0.00505 |
| BNIPL | 0.02922 | HIC1 | 0.02431 | hsa-mir-4523 | 0.00285 | hsa-mir-513a-1 | 0.00044 |
| BRCA2 | 0.04905 | KCNAB1 | 0.04351 | hsa-mir-4640 | 0.02552 | hsa-mir-513a-2 | 0.01008 |
| BTBD17 | 0.00038 | KIAA1755 | 0.00097 | hsa-mir-4758 | 0.01321 | hsa-mir-670 | 0.00427 |
| BUB1 | 0.00036 | KIR2DS4 | 0.02893 | hsa-mir-4763 | 0.00482 | hsa-mir-6883 | 0.00066 |
| BUB1B | 0.04389 | KRT37 | 0.01834 | hsa-mir-516a-1 | 0.0090 | hsa-mir-770 | 0.02063 |
| C10orf91 | 0.01755 | MAN1C1 | 0.00371 | hsa-mir-516a-2 | 0.01622 | hsa-mir-7978 | 0.01744 |
| C11orf86 | 0.00210 | OPCML | 0.00031 | hsa-mir-519a-2 | 0.00037 |  |  |
| C17orf53 | 0.02319 | P2RY14 | 0.00555 | hsa-mir-522 | 0.01996 |  |  |
| CALHM3 | 0.02035 | PAMR1 | 0.01947 | hsa-mir-548d-1 | 0.01670 |  |  |
| CAMKV | 0.00009 | PIP | 0.0036 | hsa-mir-548f-1 | 0.00848 |  |  |
| CCDC185 | 0.00213 | PPIAP45 | 0.01262 | hsa-mir-548k | 0.01787 |  |  |
| CCNE1 | 0.00002 | PTPRN2 | 0.02962 | hsa-mir-551b | 0.00407 |  |  |
| CCNF | 0.02665 | RASSF2 | 0.01618 | hsa-mir-5579 | 0.01616 |  |  |
| CD177 | 0.0180 | RIPOR2 | 0.03998 | hsa-mir-588 | 0.03993 |  |  |
| CDC25C | 0.03231 | SEL1L2 | 0.02944 | hsa-mir-6728 | 0.01160 |  |  |
| CDC45 | 0.02526 | SERTM1 | 0.00946 | hsa-mir-6783 | 0.03457 |  |  |
| CDCA5 | 0.01429 | SGCZ | 0.04658 | hsa-mir-7110 | 0.00611 |  |  |
| CDCA8 | 0.00976 | SVEP1 | 0.03579 | hsa-mir-7112 | 0.02105 |  |  |
| CDH18 | 0.0099 | SYNPO2 | 0.04275 | hsa-mir-7974 | 0.02796 |  |  |
| CDKN2A | 0.00003 | TTC4P1 | 0.01830 | hsa-mir-877 | 0.00337 |  |  |
| CDKN2A-AS1 | 0.00131 | TUBB8P4 | 0.02118 | hsa-mir-93 | 0.00194 |  |  |
| CENPA | 0.00145 |  |  | hsa-mir-934 | 0.00842 |  |  |
| CGA | 0.00371 |  |  | hsa-mir-937 | 0.00024 |  |  |
| CHST13 | 0.00798 |  |  | hsa-mir-940 | 0.00234 |  |  |
| CILP2 | 0.00221 |  |  |  |  |  |  |
| CKAP2L | 0.00814 |  |  |  |  |  |  |
| CLDN19 | 0.04314 |  |  |  |  |  |  |
| CLDN6 | 0.00114 |  |  |  |  |  |  |
| CLDN9 | 0.00039 |  |  |  |  |  |  |
| COL25A1 | 0.00079 |  |  |  |  |  |  |
| COL9A2 | 0.00548 |  |  |  |  |  |  |
| CPA4 | 0.02782 |  |  |  |  |  |  |
| CRABP1 | 0.00287 |  |  |  |  |  |  |
| CRNN | 0.01092 |  |  |  |  |  |  |
| CRYBG2 | 0.00003 |  |  |  |  |  |  |
| CTCFL | 0.02811 |  |  |  |  |  |  |
| DDC | 0.00346 |  |  |  |  |  |  |
| DEPDC1 | 0.02111 |  |  |  |  |  |  |
| DEPDC1B | 0.00286 |  |  |  |  |  |  |
| DGCR9 | 0.0099 |  |  |  |  |  |  |
| DGKK | 0.01211 |  |  |  |  |  |  |
| DHRS2 | 0.00187 |  |  |  |  |  |  |
| DLGAP3 | 0.0193 |  |  |  |  |  |  |
| DLX1 | 0.01821 |  |  |  |  |  |  |
| DMBX1 | 0.01274 |  |  |  |  |  |  |
| DMRT1 | 0.00620 |  |  |  |  |  |  |
| DMRT3 | 0.00055 |  |  |  |  |  |  |
| DNMT3B | 0.03376 |  |  |  |  |  |  |
| DPYSL5 | 0.02085 |  |  |  |  |  |  |
| DUSP9 | 0.00003 |  |  |  |  |  |  |
| E2F1 | 0.00008 |  |  |  |  |  |  |
| ECE2 | 0.00209 |  |  |  |  |  |  |
| EIF4EBP1 | 0.04816 |  |  |  |  |  |  |
| EME1 | 0.00337 |  |  |  |  |  |  |
| EMX1 | 0.03575 |  |  |  |  |  |  |
| EN1 | 0.00108 |  |  |  |  |  |  |
| ERCC6L | 0.00386 |  |  |  |  |  |  |
| ESPL1 | 0.00022 |  |  |  |  |  |  |
| ESRP1 | 0.00315 |  |  |  |  |  |  |
| ESRRB | 0.02908 |  |  |  |  |  |  |
| EZH2 | 0.01715 |  |  |  |  |  |  |
| FABP7 | 0.00954 |  |  |  |  |  |  |
| FADS6 | 0.04633 |  |  |  |  |  |  |
| FAM72B | 0.00190 |  |  |  |  |  |  |
| FAM83A | 0.00499 |  |  |  |  |  |  |
| FAM83D | 0.00138 |  |  |  |  |  |  |
| FANCA | 0.04910 |  |  |  |  |  |  |
| FANCD2 | 0.00496 |  |  |  |  |  |  |
| FIRRE | 0.04151 |  |  |  |  |  |  |
| FOXM1 | 0.02587 |  |  |  |  |  |  |
| GAL | 0.00281 |  |  |  |  |  |  |
| GCGR | 0.00580 |  |  |  |  |  |  |
| GFRA4 | 0.00547 |  |  |  |  |  |  |
| GGH | 0.00138 |  |  |  |  |  |  |
| GINS4 | 0.02005 |  |  |  |  |  |  |
| GLDC | 0.00390 |  |  |  |  |  |  |
| GPRIN2 | 0.00001 |  |  |  |  |  |  |
| HJURP | 0.01588 |  |  |  |  |  |  |
| HMGA1 | 0.00146 |  |  |  |  |  |  |
| HMMR | 0.00909 |  |  |  |  |  |  |
| HRASLS | 0.01642 |  |  |  |  |  |  |
| HTR3A | 0.00029 |  |  |  |  |  |  |
| HTR6 | 0.00497 |  |  |  |  |  |  |
| IGHEP1 | 0.03171 |  |  |  |  |  |  |
| IGSF23 | 0.02147 |  |  |  |  |  |  |
| IGSF9 | 0.03967 |  |  |  |  |  |  |
| IL1RN | 0.02796 |  |  |  |  |  |  |
| IL36RN | 0.01042 |  |  |  |  |  |  |
| INAVA | 0.00014 |  |  |  |  |  |  |
| IQGAP3 | 0.00129 |  |  |  |  |  |  |
| JPT1 | 0.01039 |  |  |  |  |  |  |
| KCNS1 | 0.00563 |  |  |  |  |  |  |
| KIAA1549L | 0.00144 |  |  |  |  |  |  |
| KIF18B | 0.01269 |  |  |  |  |  |  |
| KIF1A | 0.00176 |  |  |  |  |  |  |
| KIF23 | 0.00072 |  |  |  |  |  |  |
| KIF2C | 0.00685 |  |  |  |  |  |  |
| KIF4A | 0.02642 |  |  |  |  |  |  |
| KIFC1 | 0.01657 |  |  |  |  |  |  |
| KLC3 | 0.00101 |  |  |  |  |  |  |
| KLRG2 | 0.00000 |  |  |  |  |  |  |
| KPNA2 | 0.01523 |  |  |  |  |  |  |
| KRT15 | 0.02813 |  |  |  |  |  |  |
| KRT17 | 0.00508 |  |  |  |  |  |  |
| KRT4 | 0.02221 |  |  |  |  |  |  |
| KRT83 | 0.00029 |  |  |  |  |  |  |
| KRT8P30 | 0.03585 |  |  |  |  |  |  |
| KRT8P9 | 0.02478 |  |  |  |  |  |  |
| L1CAM | 0.00000 |  |  |  |  |  |  |
| LAMP3 | 0.00369 |  |  |  |  |  |  |
| LGALS7B | 0.00048 |  |  |  |  |  |  |
| LHFPL5 | 0.02153 |  |  |  |  |  |  |
| LIN28B | 0.00094 |  |  |  |  |  |  |
| LINC00483 | 0.01709 |  |  |  |  |  |  |
| LIPG | 0.04387 |  |  |  |  |  |  |
| LIPH | 0.02318 |  |  |  |  |  |  |
| LMO1 | 0.00036 |  |  |  |  |  |  |
| LRRC37A9P | 0.00023 |  |  |  |  |  |  |
| LY6D | 0.04156 |  |  |  |  |  |  |
| MAFA | 0.00046 |  |  |  |  |  |  |
| MAFA-AS1 | 0.0132 |  |  |  |  |  |  |
| MAGEA4 | 0.01129 |  |  |  |  |  |  |
| MAL | 0.00016 |  |  |  |  |  |  |
| MAL2-AS1 | 0.00483 |  |  |  |  |  |  |
| MAST1 | 0.00008 |  |  |  |  |  |  |
| MCM10 | 0.01093 |  |  |  |  |  |  |
| MCM2 | 0.02741 |  |  |  |  |  |  |
| MCM4 | 0.00218 |  |  |  |  |  |  |
| MCRIP2P1 | 0.00791 |  |  |  |  |  |  |
| METTL7B | 0.01196 |  |  |  |  |  |  |
| MEX3A | 0.04684 |  |  |  |  |  |  |
| MIR4635 | 0.00006 |  |  |  |  |  |  |
| MS4A15 | 0.01939 |  |  |  |  |  |  |
| MYADML2 | 0.01416 |  |  |  |  |  |  |
| MYBL2 | 0.00181 |  |  |  |  |  |  |
| MYH7 | 0.00212 |  |  |  |  |  |  |
| NCAPG | 0.03381 |  |  |  |  |  |  |
| NCAPH | 0.03114 |  |  |  |  |  |  |
| NCCRP1 | 0.00219 |  |  |  |  |  |  |
| NEIL3 | 0.03025 |  |  |  |  |  |  |
| NKAIN4 | 0.00106 |  |  |  |  |  |  |
| NKX1-2 | 0.02973 |  |  |  |  |  |  |
| NLRP2 | 0.04438 |  |  |  |  |  |  |
| NLRP3P1 | 0.02207 |  |  |  |  |  |  |
| NMU | 0.00356 |  |  |  |  |  |  |
| NOL4 | 0.0008 |  |  |  |  |  |  |
| NPBWR1 | 0.03347 |  |  |  |  |  |  |
| NPY | 0.00438 |  |  |  |  |  |  |
| NTSR1 | 0.03370 |  |  |  |  |  |  |
| NXPH4 | 0.02982 |  |  |  |  |  |  |
| OASL | 0.03778 |  |  |  |  |  |  |
| OIP5 | 0.02439 |  |  |  |  |  |  |
| OLIG3 | 0.00695 |  |  |  |  |  |  |
| ONECUT2 | 0.00993 |  |  |  |  |  |  |
| OR2B6 | 0.00493 |  |  |  |  |  |  |
| OR7E110P | 0.01178 |  |  |  |  |  |  |
| OR7E62P | 0.00037 |  |  |  |  |  |  |
| ORC1 | 0.01289 |  |  |  |  |  |  |
| ORC6 | 0.04637 |  |  |  |  |  |  |
| OTOF | 0.00565 |  |  |  |  |  |  |
| OTX1 | 0.03112 |  |  |  |  |  |  |
| PAGE2 | 0.01681 |  |  |  |  |  |  |
| PAGE2B | 0.01959 |  |  |  |  |  |  |
| PAX1 | 0.02598 |  |  |  |  |  |  |
| PCSK1 | 0.01752 |  |  |  |  |  |  |
| PDCL2 | 0.00044 |  |  |  |  |  |  |
| PGLYRP3 | 0.01674 |  |  |  |  |  |  |
| PIMREG | 0.01631 |  |  |  |  |  |  |
| PLA2G4F | 0.01012 |  |  |  |  |  |  |
| PLAC1 | 0.00017 |  |  |  |  |  |  |
| PLPP4 | 0.00444 |  |  |  |  |  |  |
| PNPLA5 | 0.00113 |  |  |  |  |  |  |
| POLQ | 0.00016 |  |  |  |  |  |  |
| POU3F3 | 0.01054 |  |  |  |  |  |  |
| PPY2P | 0.03252 |  |  |  |  |  |  |
| PRAP1 | 0.01345 |  |  |  |  |  |  |
| PRR11 | 0.00268 |  |  |  |  |  |  |
| PRSS1 | 0.01227 |  |  |  |  |  |  |
| PRSS3 | 0.03525 |  |  |  |  |  |  |
| PSAT1 | 0.00694 |  |  |  |  |  |  |
| PSRC1 | 0.00052 |  |  |  |  |  |  |
| RAB11FIP4 | 0.02051 |  |  |  |  |  |  |
| RAC3 | 0.0004 |  |  |  |  |  |  |
| RACGAP1 | 0.00723 |  |  |  |  |  |  |
| RAD51AP1 | 0.0265 |  |  |  |  |  |  |
| RAD54L | 0.02969 |  |  |  |  |  |  |
| RBP2 | 0.00036 |  |  |  |  |  |  |
| RCOR2 | 0.01750 |  |  |  |  |  |  |
| RDM1 | 0.01593 |  |  |  |  |  |  |
| RF02246 | 0.00058 |  |  |  |  |  |  |
| RF02247 | 0.00613 |  |  |  |  |  |  |
| RNF128 | 0.04547 |  |  |  |  |  |  |
| RNF2P1 | 0.00027 |  |  |  |  |  |  |
| RRM2 | 0.03021 |  |  |  |  |  |  |
| RSPO4 | 0.00016 |  |  |  |  |  |  |
| RTBDN | 0.03413 |  |  |  |  |  |  |
| RTP3 | 0.03467 |  |  |  |  |  |  |
| RXFP4 | 0.02756 |  |  |  |  |  |  |
| S100A2 | 0.01929 |  |  |  |  |  |  |
| S100A9 | 0.00615 |  |  |  |  |  |  |
| SCEL | 0.02051 |  |  |  |  |  |  |
| SELENOV | 0.02252 |  |  |  |  |  |  |
| SEPT14P12 | 0.00148 |  |  |  |  |  |  |
| SGO1 | 0.01674 |  |  |  |  |  |  |
| SHISA9 | 0.00877 |  |  |  |  |  |  |
| SIM1 | 0.02204 |  |  |  |  |  |  |
| SIM2 | 0.00339 |  |  |  |  |  |  |
| SIX1 | 0.00004 |  |  |  |  |  |  |
| SLC12A5 | 0.02733 |  |  |  |  |  |  |
| SLC16A10 | 0.00713 |  |  |  |  |  |  |
| SLC30A2 | 0.04794 |  |  |  |  |  |  |
| SLC38A8 | 0.03014 |  |  |  |  |  |  |
| SLC39A5 | 0.00003 |  |  |  |  |  |  |
| SLC6A10P | 0.01515 |  |  |  |  |  |  |
| SLC7A10 | 0.00027 |  |  |  |  |  |  |
| SLC9A2 | 0.03837 |  |  |  |  |  |  |
| SLC9A4 | 0.03411 |  |  |  |  |  |  |
| SLCO4C1 | 0.00561 |  |  |  |  |  |  |
| SLIT1 | 0.00016 |  |  |  |  |  |  |
| SNORA14B | 0.03339 |  |  |  |  |  |  |
| SOHLH1 | 0.00282 |  |  |  |  |  |  |
| SOX11 | 0.00452 |  |  |  |  |  |  |
| SPAG5 | 0.01488 |  |  |  |  |  |  |
| SPC25 | 0.00145 |  |  |  |  |  |  |
| SPRR2A | 0.03112 |  |  |  |  |  |  |
| SPRR2E | 0.02204 |  |  |  |  |  |  |
| SSC4D | 0.00005 |  |  |  |  |  |  |
| SST | 0.00923 |  |  |  |  |  |  |
| STIL | 0.02354 |  |  |  |  |  |  |
| SYNGR3 | 0.03181 |  |  |  |  |  |  |
| SYT13 | 0.01273 |  |  |  |  |  |  |
| TCAM1P | 0.01535 |  |  |  |  |  |  |
| TDRD12 | 0.00683 |  |  |  |  |  |  |
| TEX19 | 0.0404 |  |  |  |  |  |  |
| TICRR | 0.01702 |  |  |  |  |  |  |
| TK1 | 0.00243 |  |  |  |  |  |  |
| TLX1 | 0.00085 |  |  |  |  |  |  |
| TNNT1 | 0.00822 |  |  |  |  |  |  |
| TOP2A | 0.04495 |  |  |  |  |  |  |
| TPX2 | 0.00029 |  |  |  |  |  |  |
| TRAIP | 0.02759 |  |  |  |  |  |  |
| TREML2 | 0.02009 |  |  |  |  |  |  |
| TREML3P | 0.02885 |  |  |  |  |  |  |
| TRIB3 | 0.01027 |  |  |  |  |  |  |
| TRIM43 | 0.03008 |  |  |  |  |  |  |
| TRIP13 | 0.00286 |  |  |  |  |  |  |
| TRPC7 | 0.04802 |  |  |  |  |  |  |
| TRPM5 | 0.00678 |  |  |  |  |  |  |
| TTK | 0.00005 |  |  |  |  |  |  |
| TUBA1C | 0.02083 |  |  |  |  |  |  |
| TUBB4A | 0.00002 |  |  |  |  |  |  |
| UBE2C | 0.00597 |  |  |  |  |  |  |
| UCHL1 | 0.00042 |  |  |  |  |  |  |
| VGLL1 | 0.00258 |  |  |  |  |  |  |
| VSTM2B | 0.00283 |  |  |  |  |  |  |
| WFDC12 | 0.00898 |  |  |  |  |  |  |
| XKR7 | 0.00073 |  |  |  |  |  |  |
| ZIC2 | 0.04759 |  |  |  |  |  |  |
| ZNF695 | 0.00098 |  |  |  |  |  |  |
| ZNF849P | 0.04654 |  |  |  |  |  |  |
| ZYG11A | 0.01234 |  |  |  |  |  |  |

*P value was for comparation of overall survivals between high and low expression ( grouped dependent on the median ) of DEG groups in EC patients.

**Supplementary Table 2.** Top five ( sorted by P Value ) KEGG pathways and GO terms identified among DEGs associated with survival using DAVID.

| Category | Term | Count | P Value |
| --- | --- | --- | --- |
| KEGG | Cell cycle | 13 | 8.98E-08 |
|  | Oocyte meiosis | 6 | 0.01611334 |
|  | Neuroactive ligand-receptor interaction | 9 | 0.03424079 |
|  | Fanconi anemia pathway | 4 | 0.03501795 |
|  | Pancreatic cancer | 4 | 0.05819098 |
| GO_BP | Cell division | 23 | 1.21E-08 |
|  | G1/S transition of mitotic cell cycle | 12 | 3.19E-07 |
|  | DNA replication initiation | 8 | 3.46E-07 |
|  | Mitotic nuclear division | 15 | 2.01E-05 |
|  | Spindle organization | 5 | 7.35E-05 |
| GO_CC | Condensed chromosome kinetochore | 9 | 4.33E-05 |
|  | Kinesin complex | 7 | 1.26E-04 |
|  | Chromosome, centromeric region | 7 | 1.89E-04 |
|  | Midbody | 9 | 6.65E-04 |
|  | Kinetochore | 7 | 0.00125837 |
| GO_MF | DNA replication origin binding | 4 | 4.96E-04 |
|  | Microtubule binding | 11 | 0.00116836 |
|  | Microtubule motor activity | 7 | 0.00124596 |
|  | Structural molecule activity | 11 | 0.00408348 |
|  | Chromatin binding | 14 | 0.00606473 |
